# The structure of Rv3717 reveals a novel amidase from *Mycobacterium tuberculosis*

**DOI:** 10.1107/S0907444913026371

**Published:** 2013-11-19

**Authors:** Atul Kumar, Sanjiv Kumar, Dilip Kumar, Arpit Mishra, Rikeshwer P. Dewangan, Priyanka Shrivastava, Srinivasan Ramachandran, Bhupesh Taneja

**Affiliations:** a Structural Biology Unit, CSIR–IGIB, South Campus, Mathura Road, New Delhi 110 025, India

**Keywords:** Rv3717, *M. tuberculosis*, *N*-acetylmuramoyl-l-alanine amidase, SAD phasing, LC/MS analysis, α/β-fold

## Abstract

Bacterial *N*-acetylmuramoyl-l-alanine amidases are cell-wall hydrolases that hydrolyze the bond between *N*-acetylmuramic acid and l-alanine in cell-wall glycopeptides. Rv3717 of *Mycobacterium tuberculosis* has been identified as a unique autolysin that lacks a cell-wall-binding domain (CBD) and its structure has been determined to 1.7 Å resolution by the Pt-­SAD phasing method. Rv3717 possesses an α/β-fold and is a zinc-dependent hydrolase. The structure reveals a short flexible hairpin turn that partially occludes the active site and may be involved in autoregulation. This type of autoregulation of activity of PG hydrolases has been observed in *Bartonella henselae* amidase (AmiB) and may be a general mechanism used by some of the redundant amidases to regulate cell-wall hydrolase activity in bacteria. Rv3717 utilizes its net positive charge for substrate binding and exhibits activity towards a broad spectrum of substrate cell walls. The enzymatic activity of Rv3717 was confirmed by isolation and identification of its enzymatic products by LC/MS. These studies indicate that Rv3717, an *N*-acetylmuramoyl-l-alanine amidase from *M. tuberculosis*, represents a new family of lytic amidases that do not have a separate CBD and are regulated conformationally.

## Abstract 2

The structure of Rv3717 determined to 1.7 Å resolution by Pt-SAD phasing reveals a unique autolysin that lacks a cell-wall-binding domain. Rv3717 utilizes its net positive charge for substrate binding and exhibits activity towards a broad spectrum of substrate cell walls. Structural analysis reveals that Rv3717 utilizes a β-hairpin turn at its N-terminus to autoregulate its enzymatic activity.

## 1. Introduction

Mycobacterial diseases such as tuberculosis and leprosy remain a great threat to human life. In 2010, approximately 8.8 million incidences of and 1.1 million deaths from tuberculosis were reported, while there were an additional 0.35 million deaths from HIV-associated tuberculosis (World Health Organization, 2011 ▶). An increase in multidrug-resistant cases in both developed and developing countries in recent years, including associated mortality in HIV co-infected patients (World Health Organization, 2011 ▶), has resulted in renewed concerted efforts to search for new drugs and drug targets to combat this pathogen (Raviglione *et al.*, 2012 ▶; Bhardwaj *et al.*, 2011 ▶). Bacterial cell-wall biogenesis and its metabolism play crucial roles in the bacterial life cycle and in events pertaining to survival and adaptability in host tissues (Kaur *et al.*, 2006 ▶). Consequently, the enzymes involved in cell-wall biosynthesis, including the biosynthesis of mycolic acid and peptidoglycans, are considered to be promising targets for newer therapeutic interventions in *Mycobacterium tuberculosis* infections (Lopez-Marin, 2012 ▶).

The mycobacterial cell envelope is a thick rigid structure consisting of an inner plasma membrane and an outer cell wall formed by a peptidoglycan–arabinogalactan complex. The outer lipid layer is esterified with mycolic acids that are covalently linked to the arabinogalactan layer (Hoffmann *et al.*, 2008 ▶; Zuber *et al.*, 2008 ▶). This arabinogalactan layer is further enclosed by a capsular layer of glycans, lipids and proteins and serves as a barrier against the penetration of antibiotics (Brennan & Nikaido, 1995 ▶). The unusual multilayer cell envelope limits the number of agents that are effective against tuberculosis and is also involved in the development of drug-resistant mycobacterial strains (McNeil & Brennan, 1991 ▶; Nguyen & Thompson, 2006 ▶; Hett & Rubin, 2008 ▶).

Most bacteria encode a large number of redundant peptido­glycan hydrolases in their genomes that hydrolyze specific bonds in the cell-wall peptidoglycan (PG). More than 30 PG hydrolases have been identified in *Escherichia coli* (van Heijenoort, 2011 ▶) and *Bacillus subtilis* (Smith *et al.*, 2000 ▶) on the basis of amino-acid sequence similarity. The PG hydrolases, also termed autolysins, are highly specific and have been broadly classified into three types on the basis of the bonds cleaved: *N*-acetylmuramoyl-l-alanine amidases, glycan strand-cleaving hydrolases and endopeptidases (Smith *et al.*, 2000 ▶; Vollmer *et al.*, 2008 ▶). PG hydrolases show a high degree of redundancy for the cleavage of specific bonds, underscoring their functional importance. PG hydrolases have important roles in cleavage of the septum of daughter cells, cell-wall turnover and even autolysis under specific conditions (Vollmer *et al.*, 2008 ▶; van Heijenoort, 2011 ▶). Relatively few PG hydrolases have been identified in mycobacteria. *M. phlei* autolysin was one of the first cell-wall hydrolases from mycobacteria to be characterized (Li *et al.*, 1999 ▶). A few other PG hydrolases, namely Rv3915 (CwlM), Rv2719c (cell-wall hydrolase), Rv2518c (l,d-transpeptidase), Rv1477 (RipA; endopeptidase) and Rv1478 (RipB; endopeptidase), have been identified in *M. tuberculosis*.

*N*-Acetylmuramoyl-l-alanine amidases belonging to the PG hydrolase family hydrolyse the amide bond between *N*-acetylmuramic acid and l-alanine. *N*-Acetylmuramoyl-l-alanine amidases play a crucial role in bacterial surface growth, sporulation, germination and cell division, and bacterial pathogenesis, including adhesion to the host cells and invasion (Allignet *et al.*, 2001 ▶; Berry *et al.*, 1989 ▶; Rupp *et al.*, 2001 ▶; Milohanic *et al.*, 2001 ▶; Loessner *et al.*, 2002 ▶; Kumar *et al.*, 2013 ▶). Rv3915 (CwlM) is the only *N*-acetylmuramoyl-l-alanine amidase identified in *M. tuberculosis* to date (Deng *et al.*, 2005 ▶) and consists of distinct PG-binding and catalytic domains. We show here that Rv3717 is an *N*-acetylmuramoyl-l-alanine amidase exhibiting cell-wall hydrolase activity and report its crystal structure at 1.7 Å resolution. Rv3717 consists of a single domain and lacks the cell-wall-binding domain (CBD) usually observed in bacterial autolysins or phage endolysins. The crystal structure suggests that the overall surface charge on Rv3717 enables it to bind and hydrolyse the cell walls of several bacteria and therefore may underlie its broad spectrum of activity. In addition, the structure reveals a short flexible hairpin turn that partially occludes the active site. This hairpin turn appears to be functionally analogous to the short helical subdomain of *Bartonella henselae* *N*-acetylmuramoyl-l-alanine amidase (AmiB; Yang *et al.*, 2012 ▶) involved in autoregulation. This type of regulation of activity of PG hydrolases may be a general mechanism used by some of the redundant amidases to regulate cell-wall hydrolase activity in bacteria.

## 2. Materials and methods

### 2.1. Cloning, expression and purification of Rv3717

The coding region of Rv3717 encoding residues 25–241 was PCR-amplified from *M. tuberculosis* H37Rv genomic DNA using forward and reverse primers. The forward primer introduced a *Bam*HI site upstream of the 25th residue, whereas the reverse primer introduced a *Xho*I site 3′ to the stop codon. The PCR product was digested with *Bam*HI and *Xho*I and cloned in a similarly digested pET-28a(+) vector to give the expression plasmid pAMI-A. The protein encoded by pAMI-A consists of 217 residues of Rv3717 at the C-terminus with an additional 34 residues at the N-terminus of the sequence consisting of a His tag and additional leading residues arising from cloning. The clone lacks the N-terminal 24 residues of Rv3717 encoding a predicted signal peptide. pAMI-A was transformed into *E. coli* C41 (DE3) strain for expression. The transformed cells were grown at 37°C to an *A*_600_ of ∼0.6 in Terrific Broth. The culture was then chilled on ice for 1 h and protein expression was induced with 0.25 m*M* IPTG (isopropyl β-d-1-thiogalactopyranoside) for 12 h with constant shaking at room temperature (25°C). Subsequently, the cells were harvested by centrifugation at 6000*g* for 10 min at 277 K. The pellet was resuspended in sonication buffer (20 m*M* Tris–HCl pH 7.9, 500 m*M* NaCl, 5 m*M* imidazole, 1 m*M* phenylmethylsulfonyl fluoride) and was incubated with 1 µg ml^−1^ lysozyme on ice for 1 h. The cells were lysed by sonication for 5 min and centrifuged at 29 000*g* for 30 min at 4°C to remove cell debris. The His-tagged protein was purified by affinity chromatography by loading the supernatant onto nickel–nitrilotriacetate (Ni–NTA) resin (Qiagen). The column was washed with wash buffer 1 (20 m*M* Tris–HCl pH 7.9, 500 m*M* NaCl) containing 5, 20, 50 and 100 m*M* imidazole in successive steps and the bound protein was eluted with 15 column volumes of elution buffer (20 m*M* Tris–HCl pH 7.9, 500 m*M* NaCl, 300 m*M* imidazole). Fractions containing significant amounts of purified protein, as determined by SDS–PAGE (12%), were pooled. Recombinant His-tagged Rv3717 thus purified from pAMI-A (rRv3717-A) was dialyzed against phosphate-buffered saline (PBS) at 4°C, concentrated to 0.50 mg ml^−1^ using an Amicon Ultra-15 (5K NMWL) concentrator (Millipore, USA) and stored at 4°C until further use. All activity assays described below were carried out using rRv3717-A.

### 2.2. Construct for crystallization experiments

pAMI-A was digested with *Bam*HI and *Xho*I and the insert was gel-purified and cloned into a similarly digested pET28-His10-Smt3 vector to give the expression vector pAMI-B. pAMI-B encodes the Rv3717 polypeptide (residues 25–241) fused to an N-terminal His_10_-Smt3 tag. The recombinant construct was transformed into *E. coli* BL21(DE3) cells and the tagged protein was purified as described previously (Kumar *et al.*, 2011 ▶). The N-terminal tag was then cleaved overnight using the Smt3-specific protease Ulp1 (at an Ulp1:protein ratio of 1:500) at 4°C. The tag and protease were removed over nickel–nitrilotriacetate resin (Qiagen). The removal of the Smt3 tag leaves one serine residue at the N-­terminus of the purified protein. The recombinant protein thus purified from pAMI-B (rRv3717-B) was dialysed in low-­salt buffer (20 m*M* HEPES pH 7.4, 50 m*M* KCl, 0.1% β-­mercaptoethanol), concentrated using Amicon Ultra 3 kDa molecular-weight cutoff filter units and stored at 4°C until further use. rRv3717-B was used in all crystallization experiments described here.

### 2.3. Crystallization of Rv3717

Crystals of wild-type rRv3717-B were obtained by the sitting-drop vapour-diffusion method by mixing 2 µl protein (13 mg ml^−1^) with 1.5 µl reservoir solution (50 m*M* Tris–HCl pH 7.5, 1.6 *M* ammonium sulfate, 10 m*M* MgCl_2_) at 25°C. Crystals appeared in 2 d and were allowed to grow for a further two weeks at the same temperature. Crystals of rRv3717-B were soaked for 2 h in 10 m*M* potassium tetrachloroplatinate (II) diluted in cryoprotectant (reservoir solution with 20% ethylene glycol) and flash-cooled in liquid nitrogen until data collection.

### 2.4. Data collection and data processing

The Pt-soaked crystal was subjected to a fluorescence scan under an attenuated beam at the Pt *K* edge on beamline BM14 at the ESRF, Grenoble and the data were analysed with *CHOOCH* (Evans & Pettifer, 2001 ▶). Diffraction data were collected from the same crystal at the Pt peak wavelength of 1.0714 Å. The data were processed using *MOSFLM* (Leslie & Powell, 2007 ▶) and were scaled using *SCALA* within the *CCP*4 package (Potterton *et al.*, 2003 ▶; Winn *et al.*, 2011 ▶). The crystal belonged to space group *I*2_1_2_1_2_1_ and contained one molecule in the crystallographic asymmetric unit. Data-collection and refinement statistics are summarized in Table 1 ▶.

### 2.5. Structure determination and refinement

The structure of Rv3717 was solved by the single-wavelength anomalous dispersion (SAD) phasing method with data collected at the peak energy of the X-ray absorption spectrum of platinum using the *Auto-Rickshaw* automated structure-determination platform (Panjikar *et al.*, 2005 ▶, 2009 ▶). Further model building of the solution obtained was carried out in *Coot* (Emsley *et al.*, 2010 ▶) and refinement was performed using *REFMAC*5 (Murshudov *et al.*, 2011 ▶) and *BUSTER* v.2.10.0 (Bricogne *et al.*, 2011 ▶) with TLS refinement (Painter & Merritt, 2006 ▶; residues 2–214). The final coordinates were validated using *MolProbity* (Chen *et al.*, 2010 ▶). Details of the model building and refinement are given in Table 2 ▶. The surface electrostatic potential was calculated using *APBS* (Baker *et al.*, 2001 ▶), while the net charge on the protein was estimated as the difference between (Lys + Arg) and (Asp + Glu) residues as described previously (Low *et al.*, 2011 ▶); the zinc ion was included in the net charge calculation with a value of *Z* = +2.

The coordinates and structure factors of Rv3717 have been deposited in the PDB with accession code 4lq6.

### 2.6. Activity of rRv3717-A

The role of rRv3717-A in ‘autolysis’ was indirectly tested by investigating the activity of the purified protein towards the mycobacterial cell wall of a live *M. smegmatis* culture. *M. smegmatis* was grown to an *A*_600_ of 1.0 and 1 ml of the culture was incubated with increasing concentrations of purified rRv3717-A (1, 25 or 50 µg protein) in 1× PBS at 37°C. The *A*_600_ was monitored for 200 min (without shaking) and the experiment was performed in triplicate. BSA (50 µg) was used as a negative protein control, PBS was used as a buffer control and lysozyme (50 µg) was used as a positive control to confirm lysis.

### 2.7. Activity assay with metal ions

The dependence of the activity of rRv3717-A on metal ions was tested using heat-killed *M. smegmatis* cells. 5 nmol purified rRv3717-A in 20 m*M* potassium phosphate buffer was incubated with 1 ml heat-killed *M. smegmatis* cells (previously grown to an *A*_600_ of 1.5–2.0 and boiled in a water bath for 30 min). The assay could not be performed by depleting the presence of residual metal ions from the protein using EDTA, as the protein precipitated during dialysis with the metal-ion chelator. Therefore, molar excesses of different metal ions (Zn^2+^, Cu^2+^, Co^2+^ and Mg^2+^) at various concentrations were used for the assay (*i.e.* 0, 1, 10, 100 and 1000 µ*M*). The density of the cell suspension was monitored every minute by the *A*_600_ for 4 h at 37°C using a Cary 100 UV–Vis spectrophoto­meter. In the same assay, 5 nmol purified lysozyme was used as a positive control and 5 nmol BSA in the absence and presence of different metal ions was used as a negative control.

### 2.8. Zymography

*In situ* zymography was performed as described previously (Jayaswal *et al.*, 1990 ▶) with minor modifications. Briefly, 12% SDS–PAGE gels were prepared containing 0.5% heat-killed cells of different bacterial species (*Paenibacillus* sp., *Bordetella avium*, *E. coli* DH5α, *Enterobacter aerogenes*, *Lactobacillus acidophilus*, *Bacillus thuringiensis*, *B. pumilus*, *B. subtilis* and *E. coli* W3110) as substrates. 4 µg (143 pmol) rRv3717-A was loaded in each lane of the gel. Following electrophoresis, the gels were washed twice in 50 ml Milli-Q water with gentle stirring for 30 min and were then incubated at 37°C with 200 ml renaturation buffer (50 m*M* Tris–HCl pH 7.9, 200 m*M* NaCl, 5 m*M* CaCl_2_, 2 µm ZnCl_2_, 1% Triton-X) for 12–16 h with gentle shaking (Buist *et al.*, 1995 ▶). The zymographs were then rinsed with Milli-Q water and the gels were stained with 0.01% KOH and 0.1% methylene blue for 30 min and washed with Milli-Q water. Lysozyme (4 µg) and bovine serum albumin (BSA; 4 µg) were used as positive and negative controls, respectively. Clear bands on an opaque background indicated hydrolysis. The molecular masses of the clarified bands were determined by comparison with pre-stained molecular-weight standards electrophoresed on the same gel.

### 2.9. LC/MS analysis of enzymatic products of the *N*-­acetylmuramoyl-l-alanine amidase activity of rRv3717-A

The activity of rRv3717-A as an *N*-acetylmuramoyl-l-alanine amidase was confirmed by LC/MS with the commercially obtained substrate *N*-acetylmuramoyl-l-alanine-d-isoglutamine hydrate (Sigma). A reaction mixture consisting of 100 µg rRv3717-A was incubated with 300 µg *N*-acetyl­muramoyl-l-alanine-d-isoglutamine hydrate at 37°C for 4 h. The reaction was terminated by precipitating the protein with 8.9:1:0.1 acetonitrile:water:formic acid on ice. The samples were centrifuged at 14 000 rev min^−1^ for 10 min to remove any residual protein and the sample was subjected to analysis on an LC/ESI-MS (Quattro Micro API, Waters) coupled to an UPLC inlet. Briefly, the supernatant was loaded onto an Acquity UPLC BEH shield RP18, 1.7 µm, 2.1 × 100 mm column comprising a mobile phase of buffer *A* (acetonitrile, 0.1% formic acid) and buffer *B* (water, 0.1% formic acid) at a flow rate of 0.1 ml min^−1^. A linear gradient of 5–20% buffer *B* was run over 5 min and the peaks were monitored at 212 nm. MS analysis was carried out with the help of an LCQDeca IonTrap Electrospray Mass Spectrometer (Thermoelectron, Waltham, Massachusetts, USA). The same amount of substrate was incubated in the reaction buffer without protein as a negative control and subjected to LC/MS analysis.

### 2.10. Docking of substrate with Rv3717

Docking of *N*-acetylmuramic acid (NAM) with Rv3717 was performed using the *PatchDock* server (Schneidman-Duhovny *et al.*, 2005 ▶). Coordinates of NAM were obtained from the complex structure of the peptidoglycan-recognition protein in complex with NAM (PDB entry 3olk; Sharma *et al.*, 2012 ▶). All parameters used for docking were the defaults of the *PatchDock* server. The best solution obtained from *PatchDock* was used for model preparation. All analysis in this study was performed using the same Rv3717–NAM complex structure. A complete peptidoglycan layer (PG) was modelled with Rv3717 using the coordinates of PG from the solution structure of cell-wall peptidoglycan (Meroueh *et al.*, 2006 ▶; coordinates received from S. Mobashery, personal communication).

## 3. Results

### 3.1. Identification and structure determination of Rv3717

Domain analysis of all of the proteins in the genome of *M. tuberculosis* H37Rv was carried out using the CDD (Marchler-Bauer *et al.*, 2011 ▶) and Pfam (Punta *et al.*, 2012 ▶) databases. Each of these searches identified two *N*-acetyl­muramoyl-l-alanine amidases in the *M. tuberculosis* genome: Rv3915, previously characterized as an *N*-acetylmuramoyl-l-alanine amidase (Deng *et al.*, 2005 ▶), and Rv3717, previously annotated as a conserved hypothetical protein. Rv3717 consists of 241 amino acids with a predicted molecular mass of 24.8 kDa. The *SignalP* server (Petersen *et al.*, 2011 ▶) predicted the protein to have a signal peptide with a cleavage site between the 24th and 25th residues. Hence, a truncated construct of Rv3717 lacking the predicted signal peptide and starting at the 25th amino-acid position from its N-terminus, comprising 218 residues (including an N-terminal serine as a cloning artefact), was expressed and purified as described in §[Sec sec2]2. The estimated net charge on the protein is *Z* = +6. Purified rRv3717-B was successfully crystallized by the sitting-drop vapour-diffusion method; crystals appeared in 2–3 d. The structure of rRv3717-B was determined to 1.7 Å resolution by the Pt-SAD phasing method using native crystals soaked with a Pt salt (as described in §[Sec sec2]2) and refined to final *R*_work_ and *R*_free_ values of 0.160 and 0.184, respectively.

### 3.2. Overall structure and role of rRv3717-­B as a functional unit

**Figure 1:**
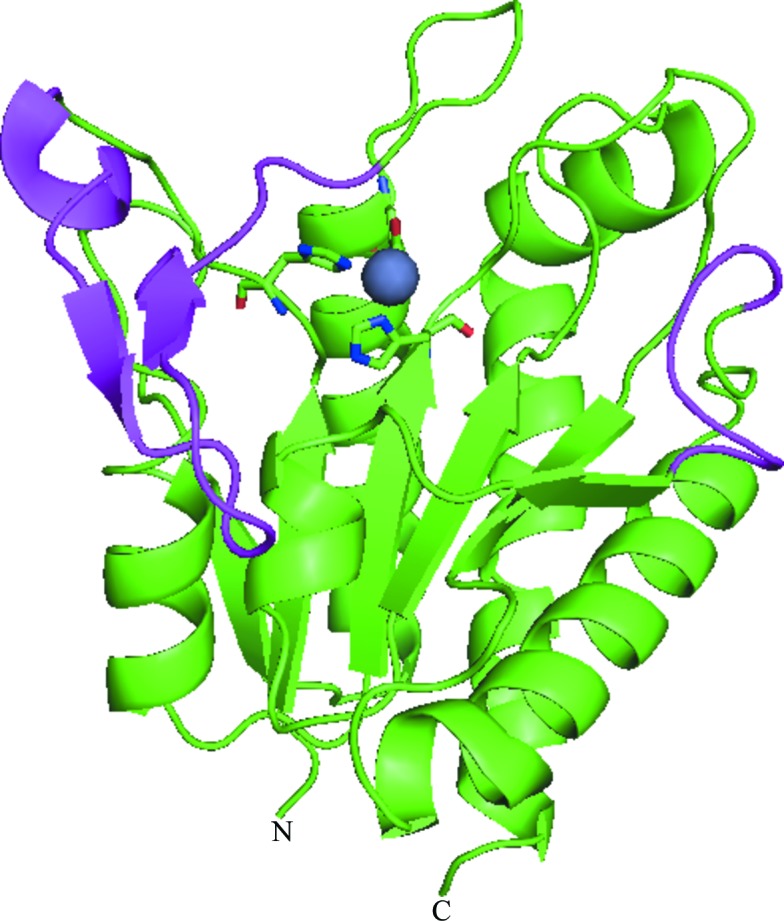
Overall structure of rRv3717-B indicating a single-domain structure for this amidase. The unique ^16^β-­hairpin^36^ and ^148^loop^154^ are shown in magenta. A bound zinc ion (sphere) marks the catalytic centre of the metalloenzyme. The N- and C-­termini are labelled.

rRv3717-B represents a unique family of amidases with a single catalytic domain with dimensions of 45 × 45 × 38 Å. There is one molecule in the asymmetric unit and the protein exists as a monomer. No separate cell-wall/substrate-binding domain (CBD) as present in other amidase crystal structures (Korndörfer *et al.*, 2006 ▶) was observed (Fig. 1 ▶).

The overall structure of rRv3717-B comprises an α/β-fold with a central six-stranded twisted β-sheet surrounded by α-­helices on both sides (Fig. 1 ▶). The final model consists of 213 residues of the molecule. Two residues at the N-terminus (including the additional serine) and three residues at the C-­terminus were disordered and could not be included in the final model. One Zn^2+^ ion was located in the electron-density map in the active site of the metalloenzyme (Fig. 1 ▶). A β-­hairpin turn comprising residues 16–36 appears to form a short N-terminal subdomain that limits access to the active site.

### 3.3. Structural comparisons

**Figure 2:**
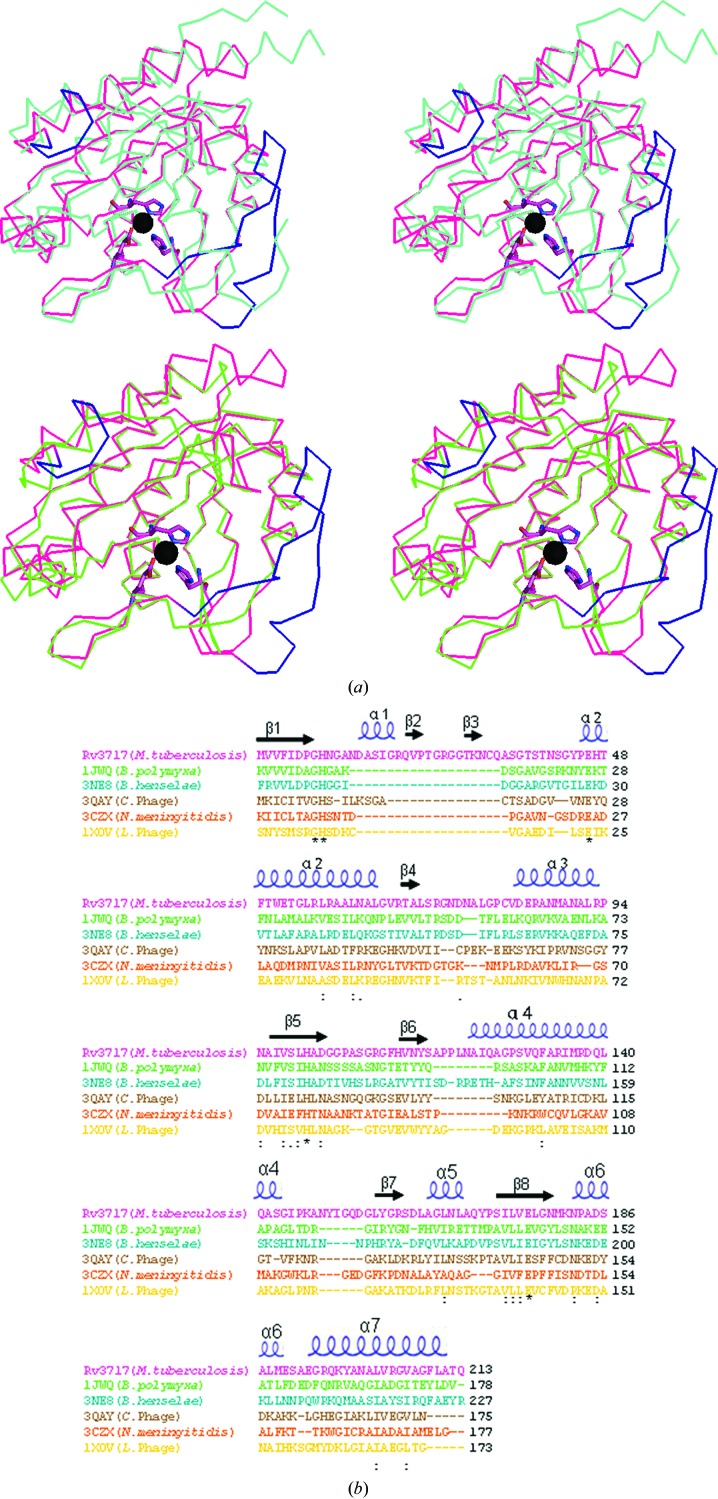
Comparative analysis of Rv3717 with other *N*-acetylmuramoyl-l-alanine amidases. (*a*) Stereoview of the superposition of rRv3717-B (magenta) with representative EADs of *N*-acetylmuramoyl-l-alanine amidases from *Bartonella henselae* (PDB entry 3ne8; cyan) or *Bacillus polymxa* (PDB entry 1jwq; green) indicates structural similarities (see text for more details). A bound zinc ion (sphere) marks the active site of Rv3717. The ^16^β-hairpin^36^ at the N-terminus and ^148^loop^154^ of Rv3717 (shown in blue) partially occlude the active site. Active-site residues in Rv3717 in coordination with Zn^2+^ are shown in stick representation. (*b*) Sequence alignment of Rv3717 and its structural homologues as identified from *DALI*. Sequences are shown in the same colours as their structural counterparts in (*a*).

A search for structural homologues of rRv3717-B was carried out in *DALI* using the final refined coordinates. The *DALI* search resulted in hits for the catalytic or enzymatically active domain (EAD) of autolysins and endolysins. The top hit for rRv3717-B in *DALI* shows a closest match to PDB entry 1jwq (T. Yamane, Y. Koyama, Y. Nojiri, T. Hikage, M. Akita, A. Suzuki, T. Shirai, F. Ise, T. Shida & J. Sekiguchi, unpublished work) annotated as an *N*-acetylmuramoyl-l-alanine amidase with a *Z*-score of 25.3 and an r.m.s. deviation of 1.7 Å for 179 C^α^ atoms, suggesting an overall similar structure and a similar amidase role for Rv3717. Other top matches were PDB entries 3ne8 (Yang *et al.*, 2012 ▶), 3qay (Mayer *et al.*, 2011 ▶), 3czx (Midwest Center for Structural Genomics, unpublished work) and 1xov (Korndörfer *et al.*, 2006 ▶), with *Z*-­scores of 24.3, 21.0, 19.7 and 18.6, respectively. These structures superpose well with rRv3717-B (Figs. 2 ▶ *a* and 2 ▶ *b*), with acceptable r.m.s. deviations of 1.6 Å (182 C^α^ atoms), 2.3 Å (169 C^α^ atoms), 2.1 Å (166 C^α^ atoms) and 2.0 Å (167  C^α^ atoms), respectively, despite low sequence similarities varying from 13% for 3czx to 26% for 3ne8. Upon closer examination, the overall topology of these structures was found to be identical to Rv3717 except for the insertion of a β-hairpin comprising residues 16–36 at the N-terminus (designated ^16^β-hairpin^36^) and an extended loop in the region of 148–154 in rRv3717-B (designated ^148^loop^154^) (Fig. 2*a* ▶). The average *B* factors of the ^16^β-hairpin^36^ (22.4 Å^2^) and ^148^loop^154^ (25.4 Å^2^) are marginally higher than rest of the protein (21.4 Å^2^), suggesting that these regions are more flexible than the rest of the protein.

**Figure 3:**
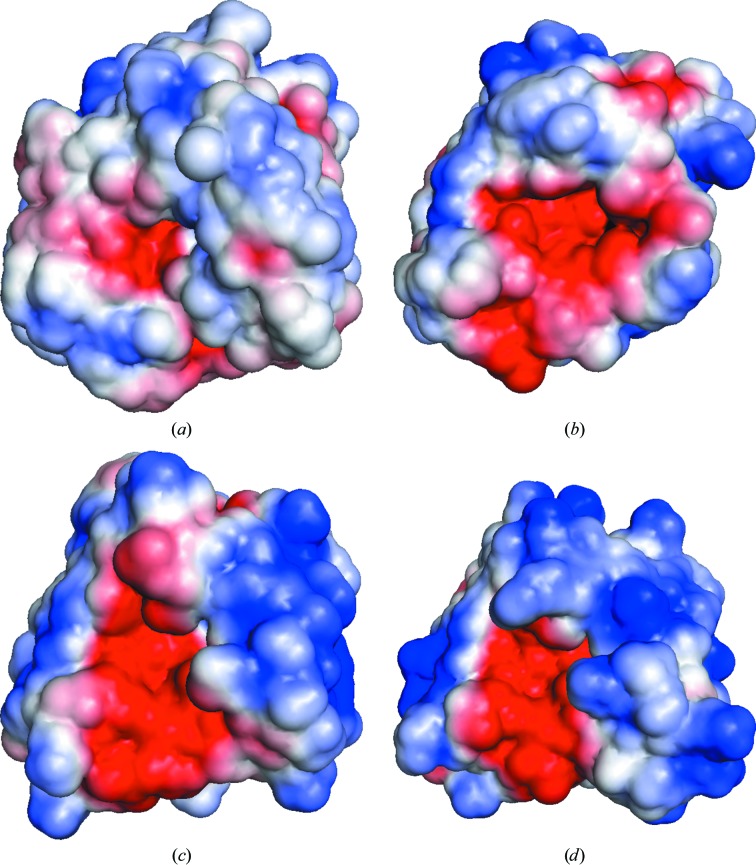
Electrostatic surface representation of Rv3717 and selected amidases. Molecular-surface representation of (*a*) rRv3717-B in the same orientation as the EADs of (*b*) CwlV (PDB entry 1jwq), (*c*) PlyPSA (PDB entry 1xov) and (*d*) CD27L (PDB entry 3qay) showing the electrostatic potential displayed on the surface. The electrostatic potential was calculated with *APBS* and is coloured with a blue to red gradient from +2.5*k*_B_ *T*/e to −2.5*k*_B_ *T*/e.

Electrostatic surface representation of amidases (Mayer *et al.*, 2011 ▶) has previously shown that while the active site is negatively charged, the surface leading to the active site is positively charged. The electrostatic surface of rRv3717-B reveals a smaller substrate-binding pocket and relatively less negative charge in comparison to other homologues (Fig. 3 ▶). The positive charge outside the binding pocket also appears to be distributed all over the surface with no distinct region with a strong positive charge. This unique charge distribution compared with other amidases suggests that the mycobacterial homologue may have evolved a somewhat different mechanism to bind and direct the PG substrate to the active site.

### 3.4. Enzymatic activity of Rv3717

**Figure 4:**
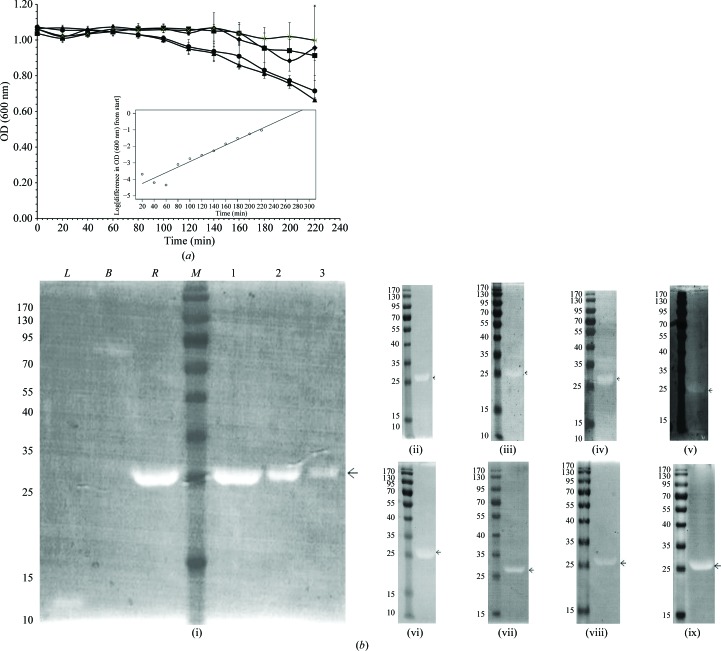
Cell-wall hydrolase activity of Rv3717. (*a*) The cell-wall hydrolase activity of rRv3717-A was monitored by incubating live *M. smegmatis* cells with no (filled squares), 1 µg ml^−1^ (crosses), 25 µg ml^−1^ (filled triangles) or 50 µg ml^−1^ (filled circles) rRv3717-A or with BSA (filled diamonds). Error bars represent one standard deviation from the mean value of OD_600_ in triplicate experiments. The inset shows the mathematical fit for rRv3717-A (50 µg ml^−1^ reaction): log(ΔOD_600_) = 0.016*x* − 4.43 [general form log(ΔOD_600_) = *ax* − *b*, where the values of *a* and *b* are average values for each point]. (*b*) Zymograph analysis of rRv3717-A with cell suspensions of different heat-killed bacteria as substrates: (i) *Paenibacillus* sp., (ii) *B. avium*, (iii) *E. coli* DH5α, (iv) *E. aerogenes*, (v) *L. acidophilus*, (vi) *B. thuringiensis*, (vii) *B. pumilus*, (viii) *B. subtilis* and (ix) *E. coli* W311. Zymograph analysis was carried out in a dose-dependent manner; in (i) lane 1 contains 4 µg, lane 2 contains 2 µg and lane 3 contains 1 µg rRv3717-A. L, Lysozyme (4 µg); B, BSA (4 µg); and R, rRv3717-A (4 µg). Lower doses are not indicated in the other panels for clarity. The zone of clearance indicates cell-wall hydrolysis. A molecular-weight marker (lane *M*) is included in each panel and is labelled in kDa.

The activity of the single catalytic domain of rRv3717-A was tested by monitoring the decrease in turbidity of a live *M. smegmatis* suspension as a substrate. Significant lytic activity was observed at 25 and 50 µg rRv3717-A per millilitre of culture (Fig. 4 ▶ *a*). In comparison, buffer control (no protein) or incubation with BSA resulted in no hydrolysis. The activity of lysozyme towards live *M. smegmatis* culture was also calculated. The activity of rRv3717-A obtained from this assay was 20.3583 × 10^−4^ AU nmol^−1^ min^−1^ and was found to be nearly 2.5-fold higher than that of lysozyme (8.1714 × 10^−4^ AU nmol^−1^ min^−1^).

The role of rRv3717-A as a general amidase was investigated by zymography using the cell walls of different bacteria (Fig. 4 ▶ *b*). rRv3717-A was able to hydrolyse the cell walls of several bacterial species in a dose-dependent manner (*i.e.* *Paenibacillus* sp., *B. avium*, *E. coli* DH5α, *E. aerogenes*, *L. acidophilus*, *B. thuringiensis*, *B. pumilus*, *B. subtilis* and *E. coli* W3110), thereby showing that rRv3717-A is a cell-wall hydrolase with broad-spectrum activity.

### 3.5. The zinc-binding motif identifies the catalytic site of Rv3717

*N*-Acetylmuramoyl-l-alanine amidases are metalloenzymes with a zinc ion being coordinated in the active site of structural homologues (Korndörfer *et al.*, 2006 ▶). A metal fluorescence scan using native protein crystals gave an excitation peak at the zinc edge. However, the peak could not unambiguously be assigned to zinc from protein crystals as a peak was also observed in a fluorescence scan with buffer controls. The zinc ion in the active site of rRv3717-B was confirmed by its trigonal bipyramidal coordination geometry in accordance with previously described coordination geometries of Zn^2+^ (Harding, 2001 ▶) as well as by phased anomalous difference maps. The Zn atom in the crystal structure was found to be coordinated to His11, Glu46 and His101. In the vicinity, three water molecules are also present to complete its coordination state. This coordination geometry for Zn^2+^ matches very well with the previously described geometries for Zn^2+^ in the active site of the structural homologues described above (Korndörfer *et al.*, 2006 ▶; Mayer *et al.*, 2011 ▶; Yang *et al.*, 2012 ▶). In addition, the average *B* factor for the metal ion refined as Zn^2+^ (18.5 Å^2^) was found to be close to those of the surrounding residues, confirming it as Zn^2+^ in the active site of Rv3717. Intriguingly, His11, His101 and Glu46 in the catalytic pocket are among the only conserved residues in the sequence alignment of Rv3717 with its structural homologues (Fig. 2*b* ▶), emphasizing the role of these conserved residues in zinc binding and catalysis despite significant sequence divergence. Gly10, which has a structural role, and Glu176, which indirectly interacts with Zn^2+^ through a water-mediated inter­action, are the only other conserved residues in the sequence alignment.

**Figure 5:**
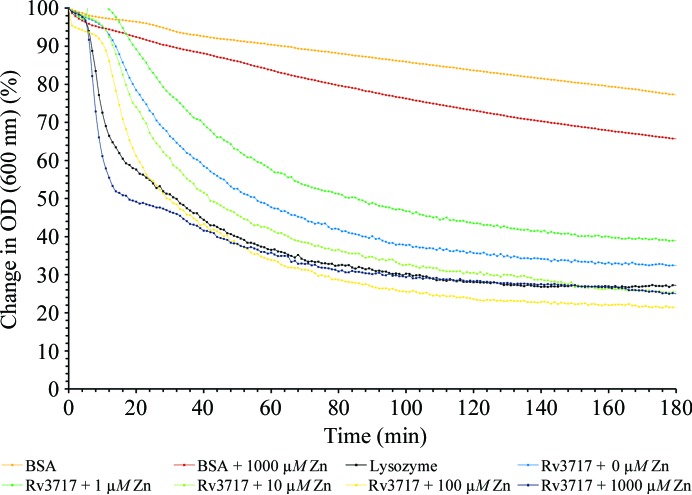
Effect of metal ions on the activity of rRv3717-A. Activity of rRv3717-A towards heat-killed *M. smegmatis* estimated with no (blue), 1 µm (sea green), 10 µm (light green), 100 µm (yellow) or 1000 µm (brown) Zn^2+^ at 37°C. BSA (orange) or BSA + 1000 µm Zn^2+^ (red) were used as negative controls, while lysozyme (black) served as a positive control for the reaction.

The zinc ion seems to bind preferentially to the protein, as no extraneous Zn^2+^ was added either in protein preparation or during crystallization. The intrinsically bound Zn^2+^ ion also enabled hydrolytic activity towards heat-killed *M. smegmatis* cells even when no additional Zn^2+^ was added to the reaction mixture (Fig. 5 ▶). The lytic activity increased marginally with increasing amounts of Zn^2+^. Despite the apparent preference for Zn^2+^ binding, several other divalent ions that were tested (Cu^2+^, Co^2+^ and Mg^2+^) appeared to substitute for Zn^2+^ and achieved similar levels of lytic activity with increasing concentrations of the respective metal ion as observed for Zn^2+^ (Supplementary Fig. S1^1^).

### 3.6. Analysis of enzymatic products of Rv3717 activity by LC/MS

**Figure 6:**
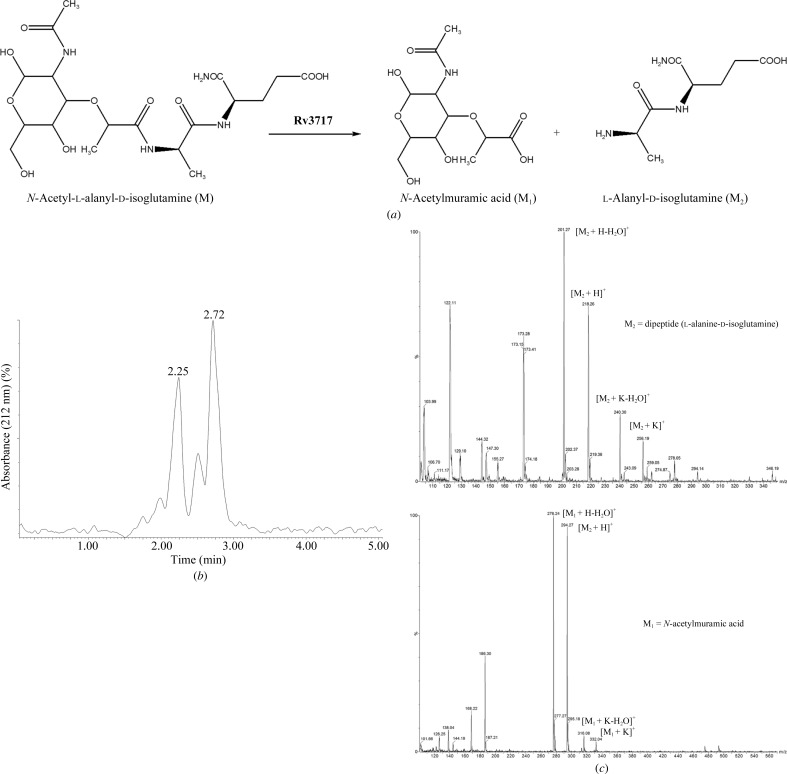
LC/MS analysis. (*a*) Schematic representation of the enzymatic reaction of Rv3717 towards *N*-acetylmuramoyl-l-alanyl-d-isoglutamine (M) resulting in products M_1_ (muramic acid; molecular mass 293.27 Da) and M_2_ (l-alanyl-d-isoglutamine (molecular mass 217.26 Da), which were analyzed by LC/MS. (*b*) The reaction mixture was purified by ultrahigh-pressure liquid chromatography (UPLC) into separate peaks of l-alanyl-d-isoglutamine (2.25 min) and muramic acid (2.72 min). (*c*) MS analysis of the UPLC-separated peaks corresponding to l-alanyl-d-isoglutamine (2.25 min; top panel) and muramic acid (2.72 min; bottom panel).

The enzymatic products obtained from the activity of rRv3717-A were analyzed to confirm the role of the enzyme as an amidase using *N*-acetylmuramoyl-l-alanyl-d-isoglutamate as a substrate (Fig. 6 ▶ *a*). Upon digestion by rRv3717-A, two peaks corresponding to the digested products l-alanyl-d-isoglutamine (2.25 min) and *N*-acetylmuramic acid (2.72 min) were obtained from HPLC separation (Fig. 6 ▶ *b*). Mass-spectrometric analysis confirmed the 2.25 min peak to be l-alanyl-d-isoglutamine (*m*/*z* = 218.26 ) and the 2.72 min peak to be *N*-­acetylmuramic acid (*m*/*z* = 294.27) (Fig. 6 ▶ *c*). None of the other potential cleavage products *N*-acetylmuramoyl-l-­alanine, l-alanine or d-isoglutamate were observed, suggesting the highly specific nature of catalysis of rRv3717-A. Furthermore, MS/MS analysis of the substrate and products was performed to confirm the identity of the masses obtained in the spectrum (Supplementary Fig. S2). In addition, a control reaction with no protein but only *N*-acetylmuramoyl-l-alanyl-d-isoglutamate in the reaction mixture yielded only the original substrate with an *m*/*z* of 493.06. No detectable masses for d-isoglutamine, l-­alanine or *N*-acetylmuramoyl-l-alanine were identified, confirming that the products result from enzymatic activity of rRv3717-A and not from spontaneous or random hydrolysis (Supplementary Fig. S3).

### 3.7. Modelling of Rv3717 with cell-wall peptidoglycan

**Figure 7:**
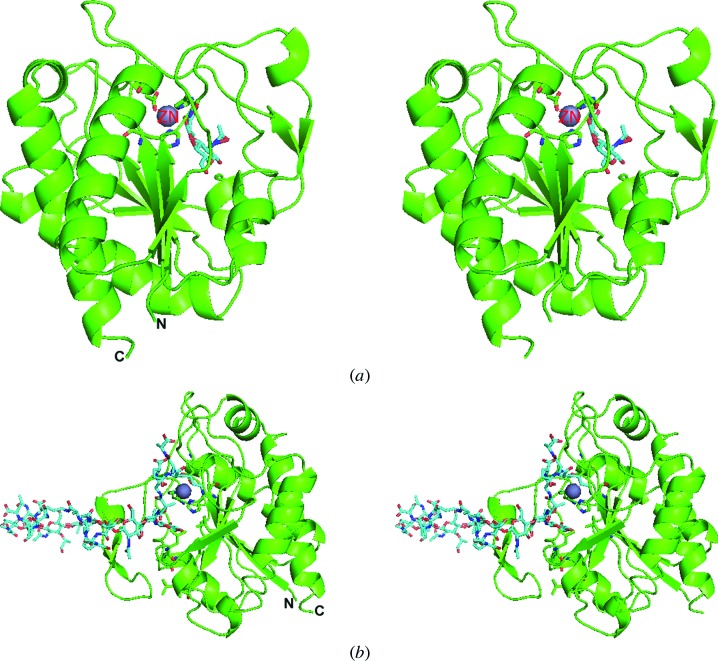
Proposed model of Rv3717 with cell-wall peptidoglycan. (*a*) Stereoview of Rv3717 in complex with *N*-acetylmuramic acid as obtained from the *PatchDock* server. (*b*) Stereoview of a proposed model of Rv3717 with cell-wall peptidoglycan. The hydrophobic residues that may be involved in PG binding as well as the catalytic residues of Rv3717 are shown in stick representation.

In order to gain insights into the mode of PG binding to rRv3717-B, a blind docking experiment using the *PatchDock* server (Schneidman-Duhovny *et al.*, 2005 ▶) was first carried out using *N*-acetylmuramic acid (NAM) as a potential ligand. The top results from the server revealed that NAM is bound in the active site of rRv3717-B close to the zinc metal ion on one side and a hydrophobic region on the other, thereby confirming the zinc-binding pocket as the substrate-binding site (Fig. 7 ▶ *a*). Zinc is present on one of the walls of the active site, allowing substrate to bind in this pocket. This docked position of NAM obtained from the *PatchDock* server was used to model the interactions of Rv3717 with the peptidoglycan layer (Fig. 7 ▶ *b*). The electrostatic surface of the protein showed that the catalytic centre where NAM is present is rich in negative charge (Fig. 3 ▶) and is surrounded by a hydrophobic surface, suggesting that the PG substrate binds in the pocket near to the zinc motif through hydrophobic interactions. The labile amide bond on the PG layer is pushed into the electronegative active site where catalysis takes place through a proton-relay system.

The active site of Rv3717 is partially occluded by a β-­hairpin insertion in the N-terminus of rRv3717-B and may restrict the access of substrate to the catalytic centre from one side. We looked at other potential interactions when the amidase comes into contact with the substrate PG layer and found that the nature of several conserved residues of other amidases is modified in Rv3717. For instance, Leu98 of CD27L (Mayer *et al.*, 2011 ▶) is a conserved hydrophobic residue: it is Trp in PlyPSA (Korndörfer *et al.*, 2006 ▶) and Tyr in AmiB (Yang *et al.*, 2012 ▶) and CwlV (Shida *et al.*, 2001 ▶). However, this position is occupied by Asn114 in Rv3717, suggesting that conservation of a hydrophobic residue at this position is not necessary for the activity of Rv3717. Additional conserved residues suggested to be involved in substrate binding, namely Leu130, Tyr131 and Ile132 of the *Clostridium difficile* amidase CD27L (Mayer *et al.*, 2011 ▶), are Leu127, Arg128 and Phe129 in PlyPSA (Korndörfer *et al.*, 2006 ▶), Phe128, His129 and Val130 in CwlV (PDB entry 1jwq) and Phe351, Gln352 and Val353 in AmiB (PDB entry 3ne8). These residues are Leu162, Ala163 and Gly164 in Rv3717 and are aligned for potential interactions with PG in the model (Fig. 7 ▶ *b*). The less bulky side chains in Rv3717 in this region appear to enlarge the substrate-binding cavity and suggest that Rv3717 may have evolved independent and altered modes of substrate recognition and binding.

## 4. Discussion

Rv3717 is a unique autolysin as it lacks the cell-wall-binding domain (CBD) that has the propensity to bind to fibronectin and laminin (Kumar *et al.*, 2013 ▶). The cell-wall hydrolases of various phages are known to exhibit high specificity towards the cell walls of host bacteria. This specificity is rendered to the hydrolase by unique structural elements in the CBD independently of the catalytic activity. In the absence of a CBD, it remains intriguing how Rv3717 binds to a wide spectrum of substrates to perform its role in cell-wall hydrolysis. The cell walls of bacteria carry a weak negative charge and the binding of the substrate cell wall is thus affected by the net charge on the CBD (Low *et al.*, 2011 ▶). In the absence of the CBD, the catalytic domain of *B. anthracis* amidase, PlyL, shows high lytic activity towards a broader host range than the full-length protein (Low *et al.*, 2005 ▶). This ability of the EAD of PlyL to be catalytically active has been postulated to be a consequence of the net positive charge of *Z* = +6 on the protein. Engineering of net charge on the catalytic domain of XlyA, a *B. subtilis* amidase, from *Z* = −3 to *Z* = +3 converts the EAD from an inactive to an active state (Low *et al.*, 2011 ▶), confirming that EADs with low net positive charge are catalytically active on their own.

Rv3717 thus appears to belong to a new subclass of autolysins that have evolved without a CBD and perform their function independently in a nonspecific manner. The single EAD domain of Rv3717 overcomes the absence of a CBD through a net positive charge of *Z* = +6 on the protein. The absence of a CBD, however, may lead to low processivity of the enzyme upon cleavage of the dipeptide and release of the substrate. Rv3717 appears to counter this by having a relatively more hydrophobic surface compared with other hydrolases (Fig. 3 ▶). The distributed positive charge on the surface of Rv3717 enables interactions with multiple regions of the substrate cell-wall peptidoglycan. These adaptations appear to help the enzyme perform its function in an effective manner, as shown by the nearly 2.5-fold higher specific activity of Rv3717 compared with lysozyme. In addition, the model of Rv3717 with peptidoglycan helps to identify several residues involved in hydrophobic interactions with the substrate to carry out its function. Conformational changes in Rv3717 upon substrate binding may lead to changes in the surface to enable more residues to interact with the substrate cell wall during the catalytic cycle.

Apart from the utilization of a net positive charge in substrate binding, Rv3717 appears to have evolved a mechanism of autoregulation through a unique short hairpin loop that is absent in other homologues. An autoregulatory mechanism has been observed in a few other PG hydrolases [*e.g. M. tuberculosis* RipA (Ruggiero *et al.*, 2010 ▶; Böth *et al.*, 2011 ▶), *Staphylococcus aureus* LytM (Odintsov *et al.*, 2004 ▶) and *Streptococcus pneumoniae* LytC (Pérez-Dorado *et al.*, 2010 ▶)]. An autoregulatory mechanism through conformational changes has also been observed in *N*-acetylmuramoyl-l-alanine amidases of both the Amidase_2 (comprising AmiD and AmpD; Carrasco-Lopez *et al.*, 2011 ▶) and the Amidase_3 (comprising AmiA, AmiB and AmiC; van Heijenoort, 2011 ▶) families. However, both these families differ from each other in sequence and structure (Vollmer *et al.*, 2008 ▶).

The autoregulatory amphipathic helix of AmiB is present in an analogous position to the autoregulatory β-hairpin of Rv3717. Asp259 of the autoregulatory helix makes direct interactions with the zinc ion in AmiB, while another residue, Asp290, extends into the active site, thereby occluding the active site. Unlike the α-helix of AmiB, however, the β-hairpin of Rv3717 appears to be in an open conformation as it does not make any interactions with Zn^2+^ or the active-site residues. Moreover, NAM in our protein–ligand model (Fig. 7 ▶ *a*) binds in the position occupied by the helix of AmiB. Rv3717 contains another short loop, comprising residues Ala148, Asn149, Tyr150, Ile151, Gly152, Gln153 and Asp154, that was not observed in its structural homologues. This loop is present in an equivalent position to that occupied by Asn343 and Asn344 of AmiB. In the AmiB structure Asn344 is positioned to make potential interactions with the occluding helix through water-mediated interactions. It is provocative to suggest a similar critical role in autoregulation for the ^148^loop^154^ in Rv3717. Although no clear interactions are observed for either the β-hairpin or the ^148^loop^154^ in the present ‘open’ conformation of Rv3717, they may be possible in its ‘closed’ state to regulate activity during different growth stages of the mycobacterium.

## 5. Conclusion

In conclusion, this is the first report of the structure of an *N*-­acetylmuramoyl-l-alanine amidase from *M. tuberculosis*. Rv3717 represents a new family of lytic amidases that do not have a separate CBD and are regulated conformationally, analogous to the cell-division amidase AmiB (Yang *et al.*, 2012 ▶). The enzymatic products of Rv3717 were analyzed by LC/MS to confirm its activity. A net positive charge on the protein, combined with the absence of a CBD, enables broad substrate specificity for Rv3717, as shown by zymography using the cell walls of several heat-killed bacteria. The surface representations suggest that the rim of the binding pocket of the enzyme is hydrophobic, in contrast to the available structures from other eubacteria. Whether this difference in surface charge is an artefact of local conformations of the apo enzyme in the ‘open’ conformation or whether the mycobacterial enzyme has evolved for the recognition of specific cell membranes of a few bacteria needs to be investigated further.

## Supplementary Material

PDB reference: Rv3717, 4lq6

Supplementary material file. DOI: 10.1107/S0907444913026371/lv5048sup1.pdf

## Figures and Tables

**Table 1:** Data-collection statistics for the crystal structure of Rv3717 Values in parentheses are for the outermost shell.

Space group	*I*2_1_2_1_2_1_
Unit-cell parameters (Å)	*a* = 37.35, *b* = 103.78, *c* = 129.97
Wavelength (Å)	1.0714
Resolution (Å)	30.91–1.68 (1.77–1.68)
*R*_merge_ † (%)	5.4 (29.1)
*R*_p.i.m._ ‡	0.026 (0.149)
*R*_anom_ §	0.035 (0.109)
〈*I*/σ(*I*)〉	25.1 (7.3)
Average multiplicity	9.4 (9.0)
Completeness (%)	100 (99.9)
Anomalous completeness (%)	100 (99.8)

† *R*_merge_ = 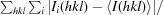 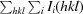, where 〈*I*(*hkl*)〉 is the mean intensity of symmetry-related reflections *I_i_*(*hkl*).

‡ *R*_p.i.m._ = 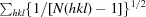 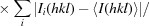 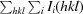.

§ *R*_anom_ = 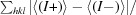 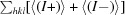.

**Table 2:** Model-refinement statistics Values in parentheses are for the highest resolution shell.

Resolution (Å)	29.52–1.68 (1.74–1.68)
Protein residues built/total residues	213/216
Total protein atoms	1620
Total waters	191
*R*_work_ †	0.160 (0.170)
*R*_free_ ‡	0.184 (0.193)
Average *B* factors (Å^2^)	
Protein atoms	19.9
Water molecules	36.9
Wilson *B* factor (Å^2^)	21.5
R.m.s.d. from ideal values§	
Bond lengths (Å)	0.010
Bond angles (°)	1.0
Ramachandran plot¶, residues in	
Most favoured region (%)	97.63
Disallowed region (%)	0.0
PDB code	4lq6

† *R*_work_ = 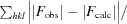 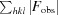, where |*F*_obs_| is the observed structure-factor amplitude and |*F*_calc_| is the calculated structure-factor amplitude.

‡ *R*_free_ is the *R* factor based on 5% of the data that were excluded from refinement.

§ As per the standard amino-acid dictionary of Engh & Huber (1991 ▶).

¶ From the *MolProbity* server (Chen *et al.*, 2010 ▶).

## Footnotes

1 Supplementary material has been deposited in the IUCr electronic archive (Reference: LV5048).
