# Supplementary material for: The structure of Rv3717 reveals a novel amidase from Mycobacterium tuberculosis
Source: Acta Crystallogr D Biol Crystallogr. 2013 Nov 19;69(Pt 12):2543–54. doi: 10.1107/S0907444913026371 (PMC3852659; doi:10.1107/S0907444913026371)

Crystal structure of Rv3717 reveals a novel amidase from *Mycobacterium tuberculosis*

Atul Kumar[$], Sanjiv Kumar[$], Dilip Kumar, Arpit Mishra, Rikeshwer P Dewangan, Priyanka Shrivastava, Srinivasan Ramachandran and Bhupesh Taneja*

Supplementary data

Supplementary Figure S1: Activity of rRv3717-A on heat killed *M. smegmatis* in presence of different metal ions

Activity of rRv3717-A was estimated with 0 (blue), 1(sea green), 10 (light green), 100 (yellow) or 1000 μm (brown) of (A) Cu^{2+}, (B) Co^{2+}, or (C) Mg^{2+} at 37°C. BSA (orange) or BSA+ 1000 μm of respective cation (red) was used as negative control; while lysozyme (black) served as positive control for the reaction.

A.

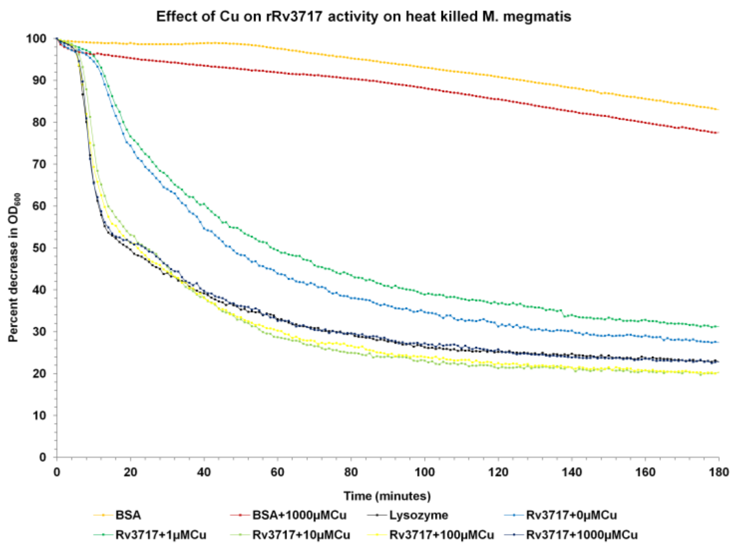

B.

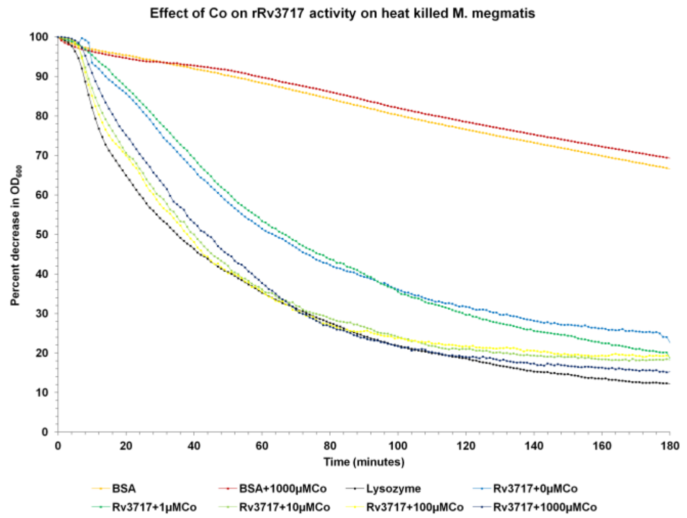
Effect of Co on rRv3717 activity on heat killed M. megmatis
Percent decrease in OD_{600}
Time (minutes)
BSA
BSA+1000µMCo
Lysozyme
Rv3717+0µMCo
Rv3717+1µMCo
Rv3717+10µMCo
Rv3717+100µMCo
Rv3717+1000µMCo

C

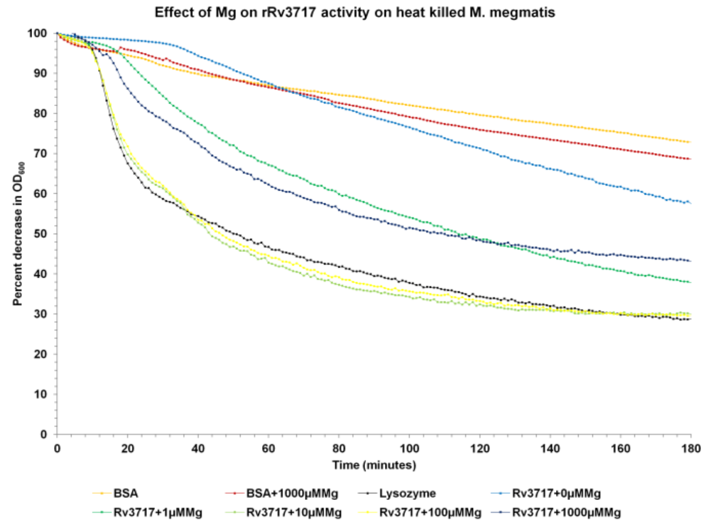
Effect of Mg on rRv3717 activity on heat killed M. megmatis
Percent decrease in OD_{600}
Time (minutes)
BSA
BSA+1000µMMg
Lysozyme
Rv3717+0µMMg
Rv3717+1µMMg
Rv3717+10µMMg
Rv3717+100µMMg
Rv3717+1000µMMg

Figure S2: MS/MS analysis of (A) substrate, N-acetylmuramoyl-L-alanyl-D-isoglutamine **(B) dipeptide,** L-alanyl-D-isoglutamine **and (C) N-acetyl muramic acid**

A

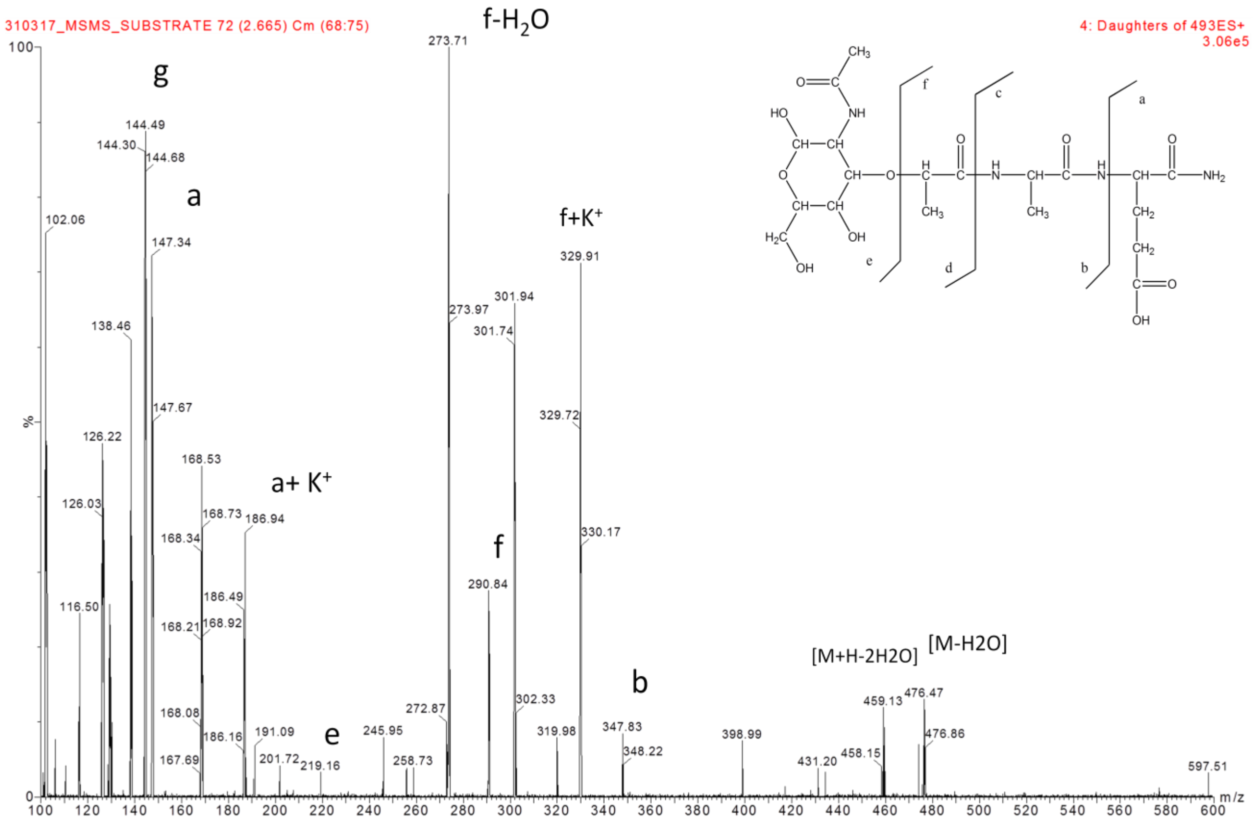

B

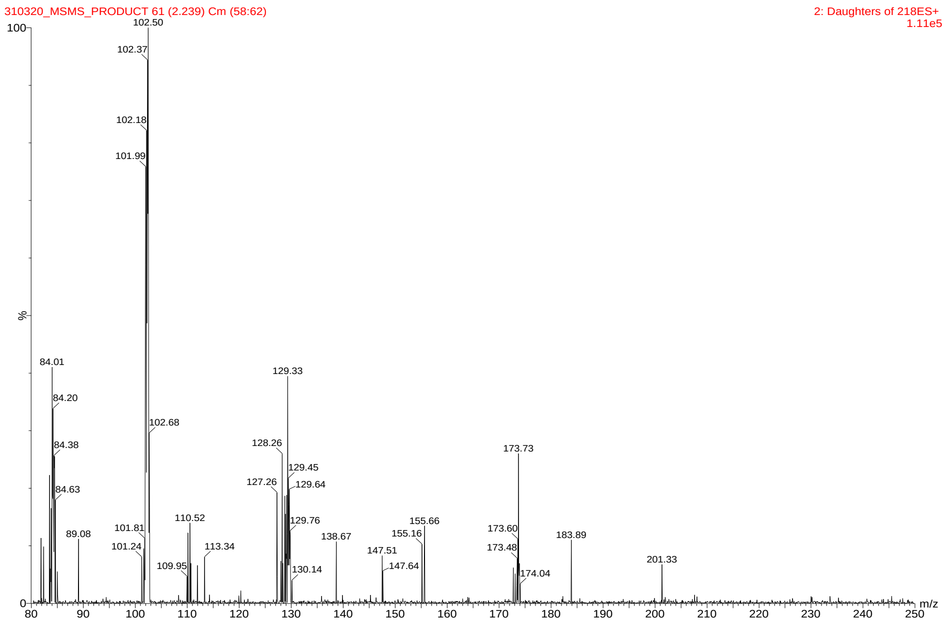
310320_MSMS_PRODUCT 61 (2.239) Cm (58:62)
2: Daughters of 218ES+
1.11e5
102.50
102.37
102.18
101.99
84.01
84.20
84.38
84.63
89.08
101.81
101.24
102.68
109.95
110.52
113.34
127.26
128.26
129.33
129.45
129.64
129.76
130.14
138.67
147.51
147.64
155.16
155.66
173.60
173.48
173.73
174.04
183.89
201.33
%
m/z

C

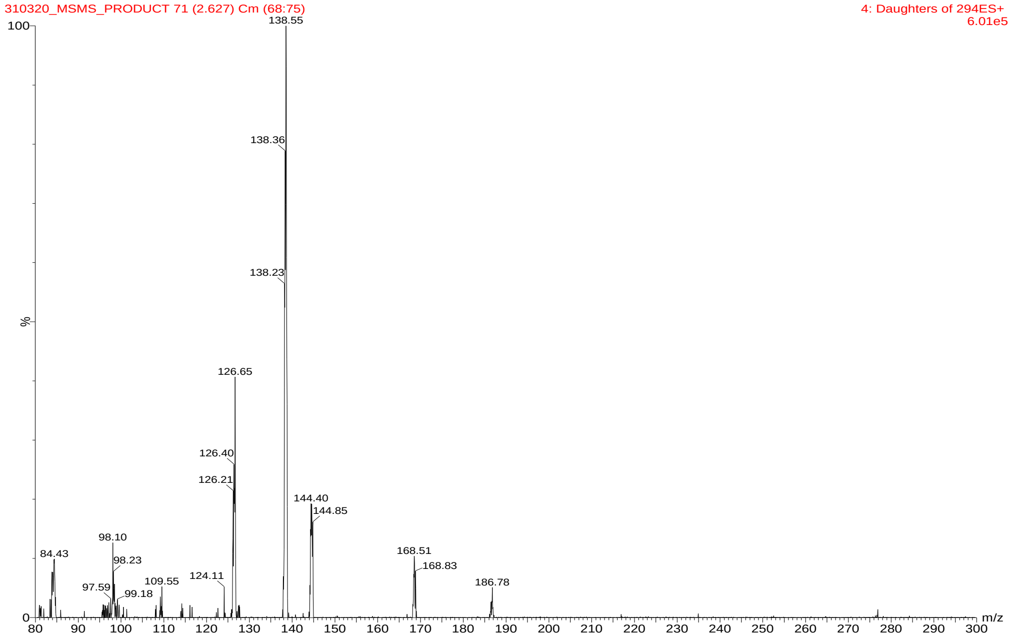
310320_MSMS_PRODUCT 71 (2.627) Cm (68:75)
4: Daughters of 294ES+
6.01e5
138.55
138.36
138.23
126.65
126.40
126.21
144.40
144.85
168.51
168.83
186.78
84.43
98.10
98.23
97.59
99.18
109.55
124.11
%
m/z

Figure S3: MS analysis of N-acetylmuramoyl-L-alanyl-D-isoglutamine in the control reaction (no protein) after 4 hours at 37ºC

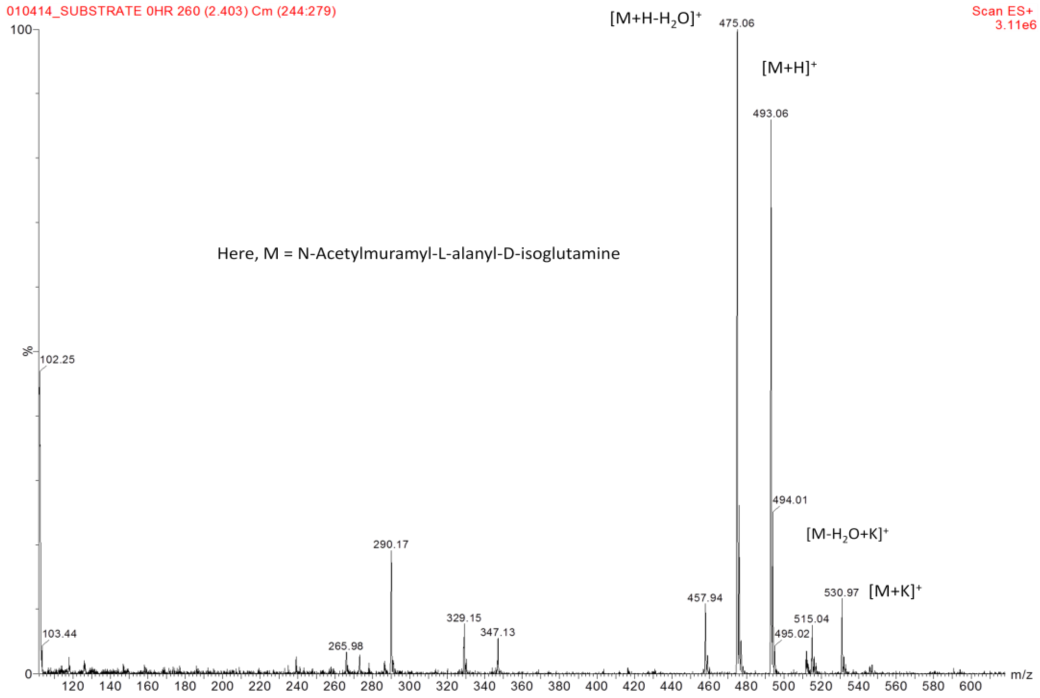

Supplement: Supplementary file 1 [file d-69-02543-sup1.pdf]
